# Optical Coherence Tomography and Optical Coherence Tomography with Angiography in Multiple Sclerosis

**DOI:** 10.3390/healthcare10081386

**Published:** 2022-07-25

**Authors:** Ioannis-Nikolaos Chalkias, Christos Bakirtzis, Demetrios Pirounides, Marina Kleopatra Boziki, Nikolaos Grigoriadis

**Affiliations:** 1 Department of Ophthalmology, AHEPA University Hospital, 54626 Thessaloniki, Greece; john_halkias@hotmail.com (I.-N.C.); dimpero@gmail.com (D.P.); 2 Multiple Sclerosis Center, Second Department of Neurology, Aristotle University of Thessaloniki, 54124 Thessaloniki, Greece; bozikim@auth.gr (M.K.B.); ngrigoriadis@auth.gr (N.G.)

**Keywords:** multiple sclerosis, optical coherence tomography, optical coherence tomography with angiography, biomarkers

## Abstract

Multiple sclerosis (MS) is an inflammatory and neurodegenerative, potentially disabling disease of the central nervous system. OCT (Optical Coherence Tomography) and OCT-A (Optical Coherence Tomography with Angiography) are imaging techniques for the retina and choroid that are used in the diagnosis and monitoring of ophthalmological conditions. Their use has recently expanded the study of several autoimmune disorders, including MS. Although their application in MS remains unclear, the results seem promising. This review aimed to provide insight into the most recent OCT and OCT-A findings in MS and may function as a reference point for future research. According to the current literature, the retinal nerve fibre layer (RNFL) and ganglion cell-inner plexiform complex (GC-IPL) are significantly reduced in people with MS and are inversely correlated with disease duration. The use of OCT might help distinguish between MS and neuromyelitis optica spectrum disorders (NMOSD), as the latter presents with more pronounced thinning in both the RNFL and GC-IPL. The OCT-A findings in MS include reduced vessel density in the macula, peripapillary area, or both, and the enlargement of the foveal avascular zone (FAZ) in the setting of optic neuritis. Additionally, OCT-A might be able to detect damage in the very early stages of the disease as well as disease progression in severe cases.

## 1. Introduction

Multiple sclerosis (MS) is a chronic autoimmune disorder of the central nervous system (CNS) characterised by inflammation, demyelination, and extensive axonal loss [1]. Although certain factors, such as Epstein–Barr virus (EBV) infection, smoking, childhood obesity, low levels of vitamin D, and ultraviolet B light (UVB) exposure, have been implicated in the pathogenesis of the disease, the definite underlying cause remains uncertain [2].

Visual symptoms and optic neuritis (ON) are the most common ocular manifestations, occurring in up to 25% of people with MS (pwMS) [3]. Post-mortem studies have shown that 90% of pwMS present degeneration and axonal loss in their optic nerves, regardless of ON history [4,5].

Given that the diagnosis of MS relies on a combination of clinical and magnetic resonance imaging (MRI) findings [6], an expensive and time-consuming method, the introduction of sensitive, low-cost biomarkers that can be used in the screening and monitoring of pwMS remains of vital importance.

The introduction of two commonly used ophthalmic imaging modalities, optical coherence tomography (OCT), and the more recent OCT with angiography (OCT-A) has revolutionised our understanding of the underlying pathophysiological mechanisms in MS, as they can indirectly provide information about the CNS. The imaging techniques OCT and OCT-A are fast, non-invasive, and inexpensive and provide anatomical and microvascular cross-sectional images of the retina, respectively. Their main application in ophthalmology is in the diagnosis and monitoring of diseases, such as age-related macular degeneration, retinal vascular disorders, and glaucoma. However, given that the retina is an extension of the brain, its use has expanded in the study of neurological and neuro-ophthalmological conditions [7].

The ganglion cells of the retina are located in the ganglion cell layer (GCL) and their axons form the retinal nerve fibre layer (RNFL). Both layers can be easily assessed using the aforementioned techniques, thus allowing the evaluation of potential neuronal and axonal degeneration in people.

This article aims to review the current OCT and OCT-A findings in pwMS and highlights the importance of ophthalmologic examination in the monitoring and progression of this disease.

## 2. Optical Coherence Tomography in Multiple Sclerosis

The imaging technique OCT was first introduced in 1991 and uses light waves to obtain cross-sectional retinal images. The RNFL and the GCL-inner plexiform layer complex (GC-IPL) are the two most commonly studied layers in MS. According to the current literature, the thickness of the RNFL and GC-IPL is found reduced in pwMS, and there is an inverse relationship with the disease duration [8,9,10]. Despite several theories, the pathophysiological mechanisms underlying these findings remain unclear [11].

Furthermore, ON is the most typical ocular manifestation, characterized by gaze-evoked retro-orbital pain and reduced visual acuity, and occurs in a significant number of pwMS [12]. Retrograde degeneration leading to axonal loss is the most probable pathological mechanism causing RNFL and GC-IPL thinning. Nevertheless, in patients with no history of ON, these findings could be either due to primary degeneration caused by the disease itself or even to retrograde degeneration after one or more subclinical episodes of ON [13].

Since MRI is the most common imaging modality used in the diagnosis of MS, many studies have focused on associating the relevant findings with those of OCT [14,15,16]. In fact, in a study by Saidha et al., GC-IPL atrophy mirrored atrophy in the white matter, brainstem, grey matter, thalamus, and the whole brain. However, a recent study by Glasner et al. suggested no correlation between white matter plaques and RNFL or GC-IPL thickness [17]. Nevertheless, grey matter atrophy seems to be the most sensitive marker of progressive MS [14]. Additionally, studies have correlated the progression of GC-IPL thinning with the development of new T2 lesions [10], damage to the optic radiations [18], and the formation of contrast-enhancing lesions [14]. One study showed that there was greater thinning in the temporal RNFL in patients with newly formed lesions in the optic radiations [19].

Several studies suggest that OCT may be used as a marker for estimating disability [20,21]. More specifically, RNFL and GC-IPL thinning were independently associated with long-term disability worsening in a recent study by Lambe et al. [22]. These findings were recently supported by Skirkova et al., who found a correlation between peripapillary RNFL values, disability, and brain MRI volumetric parameters [23]. The risk of progression can be three times higher in patients with initial RNFL thickness < 88 μm [24].

Visual acuity and contrast sensitivity are affected in patients with ON and several studies have managed to show that there are in accordance with the OCT findings [8,25,26]. GC-IPL and RNFL thinning parallel low-contrast visual acuity, and according to Lampert et al., dyschromatopsia and GC-IPL thickness are interconnected in pwMS and no history of ON [27].

Thinning of the RNFL and GC-IPL is not specific to MS; however, OCT might be helpful in differentiating it from disorders of the same spectrum. In neuromyelitis optica spectrum disorders (NMOSD), RNFL thinning tends to be more evenly distributed, whereas the temporal quadrant seems to be more affected in patients with ON MS [28]. In addition, both RNFL and GC-IPL are much more severely reduced in NMOSD than in MS. According to Brandt et al., OCT parameters may be valuable in distinguishing MS from Susac syndrome, a rare condition characterized by the triad of branch retinal artery occlusion (BRAO), encephalopathy, and hearing loss [29]. Research has shown that in patients with Susac syndrome, OCT displays scar-like pathological patterns that are strictly confined to the inner retinal layers, sparing the outer nuclear layer and the photoreceptors, thus clearly differentiating it from MS [30].

In 2008, Toledo et al. were the first to correlate OCT findings with cognitive impairment. Researchers have linked reduced RNFL thickness to cognitive disability as measured by the symbol digit modality test, a gold standard measure of information processing speed [31]. These findings were later supported by Sedighi et al., who showed that only 20% of pwMS with cognitive impairment had normal OCT findings [32]. In another study, Coric et al. noted that cognitively impaired pwMS with no history of ON had significantly reduced mean peripapillary RNFL (pRNFL) and mean macular GC-IPL (mGC-IPL) thicknesses compared to cognitively healthy pwMS [33]. Although these results may seem promising, further studies are required to support these findings.

Moreover, OCT has been used to study responses to different therapeutic options. In 2016, Pul et al. found no significant association between interferon beta (IFNβ-1b) treatment and RNFL thinning [34]. However, when Button et al. compared the effect of glatiramer acetate, natalizumab, and interferon-β-1a with the rate of GC-IPL thinning, they found that the natalizumab-treated group had a significantly lower rate of GC-IPL reduction compared to the other two [35].

Visual evoked potentials (VEP) are electrical signals generated by the visual cortex of the occipital lobe in response to visual stimuli. Klistorner et al. studied the relationship between OCT parameters and multifocal VEP (mfVEP) in patients with acute ON and found a significant reduction in RNFL thickness and mean mfVEP, particularly in the temporal quadrant [36]. Additionally, in an earlier study, researchers associated the VEP amplitude with the loss in RNFL thickness in patients with ON and noted that the main mechanism behind this loss is axonal degeneration [37].

One study examined the pupillary light response in patients with MS and found that attenuation of the melanopsin-mediated sustained pupillary constriction response was significantly associated with thinning of the GCL-IPL in pwMS with a history of ON [38].

A recent paper described peripapillary hyper-reflective ovoid mass-like structures (PHOMS) as a novel OCT finding that might be noted in people with early MS. Their presence suggests disease progression; however, more research is needed to support these results [39].

In recent years, artificial intelligence (AI) and machine learning have proven useful in medicine. In a recent study, researchers developed a system based on a convolutional neural network that can classify the disease according to the thickness of the OCT scans, thus assisting in the early diagnosis of the disorder [40]. Machine learning has also been successfully used to predict disability progression in pwMS by analysing RNFL thickness [41].

## 3. Optical Coherence Tomography with Angiography in Multiple Sclerosis

First introduced in 2014, the novel non-invasive imaging modality OCT-A complements its predecessor, OCT, by allowing the visualization of the retinal and choroidal microvasculature. Despite the need for further research for OCT-A to be integrated into clinical practice, current data suggest that it is more sensitive at the early stages of the disease, as well as detecting damage progression in advanced stages, compared to OCT. The role of hypoxia in MS has already been established [42,43], with studies observing decreased blood flow in the retinal microcirculation in people with relapsing-remitting MS [44,45]. Therefore, OCT-A, with its ability to document microvascular changes, may further supplement our understanding of this disorder.

Although more studies are needed to support these results, all current data suggest a reduced vessel density (VD) in the macular, peripapillary, or both areas in patients with MS [46,47,48,49,50]. In fact, in a large study by Murphy et al., OCT-A findings were correlated with the visual function and disability status of pwMS [46]. They also showed a significant reduction in the superficial vascular plexus (SVP) density in the eyes with MS but not in the deep vascular plexus (DVP). However, one study found significant DVP density reduction in MS compared to controls [47], while another study found a reduction only in pwMS with a history of ON, as compared to those without history of ON [48]. One recent study failed to find any significant differences in SVP VD between pwMS and controls but showed discrepancies between the two groups in the RNFL, GC-IPL, and radial peripapillary capillary (pRPC) VD [51]. Thinning of the SVP and enlargement of the FAZ appear to progress rapidly after an episode of ON and are associated with a persistent visual disability [52].

Research has also focused on measuring the VD around the optic nerve, with data suggesting a significant reduction in pwMS [53,54]. The quadrant that is most severely affected remains ambiguous, with one study suggesting the inferior and nasal quadrant [49], while another study points to the temporal quadrant [47].

The FAZ has also been used in the study of MS, and although Yilmaz et al. found no differences between the area and perimeter of the FAZ between pwMS and controls, its size was negatively related to the VD of the SVP and DVP [47].

Interestingly, in a longitudinal study with 50 stable pwMS and one-year follow-up, researchers found a slight increase in parafoveal VD, a finding that could potentially point to vascular regeneration in chronic MS, while the pRNFL and GC-IPL thickness remained unchanged [55]. However, further studies are required to support these findings.

Additionally, OCT-A has been used as a marker for disability. Higher levels of disability, as measured by the expanded disability status scale (EDSS), are related to a lower VD of the macular SVP and low-contrast visual acuity in pwMS [46]. It is worth mentioning that one study associated higher choriocapillary VDs with ongoing inflammatory disease activity [48]. Finally, Jiang et al. demonstrated lower volumetric VD (VVD), a newly developed metric system, in the retinal vascular network and DVP in pwMS with ON, and found a positive correlation between VVD of the macular SVP and EDSS and disease duration [50].

Promising results have recently emerged regarding the use of OCT-A for differentiating MS from NMOSD. There was a reduction in peripapillary and macular VD and FAZ size in people with NMOSD-ON compared to relapsing-remitting MS and controls in a study by Tiftikcioglu et al. [56]. An earlier study suggested that the best OCTA discriminant between NMOSD and MS is the VD reduction pattern, that is, the inferior to nasal (I/N) and I/T ratios for ON eyes and S/T and N/T ratios for non-ON eyes [57]. The combination of OCT and OCT-A parameters can also potentially differentiate the two conditions, as patients with NMOSD have a significantly lower average thickness of the pRNFL and GC-IPL and significantly smaller whole VD and perfusion density areas than those with MS [58].

Studies have also identified OCT-A as a potential tool for monitoring disease progression. Recent data suggest that marked vascular loss in the SVP, DVP, and pRPC may be observed in people with an initial demyelinating event, although no changes might occur in the RNFL, GC-IPL thickness, or clinical and radiological examinations [59].

Lastly, one study correlated OCT-A values with VEP measurements. They showed that there was significant agreement between low optic nerve head VD and RPC VD and prolonged VEP P100 [60].

## 4. Discussion

The OCT and OCT-A techniques are low-cost, easy-to-use, non-invasive imaging modalities that have recently expanded their use beyond their sole ophthalmologic role. As OCT has existed for more years than OCT-A, it has allowed scientists to experiment more with it and reach more conclusive results regarding the diagnosis and monitoring of MS.

In general, patients with MS exhibit RNFL and GC-IPL thinning, which are inversely related to disease duration. In ON MS, these findings are due to retrograde degeneration causing axonal loss; however, in patients with no ON history, several different theories exist. The MRI findings seem to agree with the relevant OCT values, with the grey matter being the most sensitive marker for progressive MS. Furthermore, OCT can be used to estimate the disability caused by the disease, with pwMS having thinner RNFL values initially being at an increased risk. An important distinction between MS and NMOSD is that the latter exhibited significantly lower RNFL and GC-IPL values. The OCT findings correlate with cognitive impairment, and although more data are required to support these findings, natalizumab appears to cause lower rates of GC-IPL reduction than glatiramer acetate and interferon-β-1a. Undeniably, AI and machine learning will revolutionize medicine in the upcoming years; however, currently, few studies exist, and more are needed to establish the use of OCT in predicting the risk of disability progression. 

Although OCT-A is a novel imaging technique compared to OCT, few studies have been conducted, and the results still seem promising. The advantage of OCT-A is that it can detect the disease earlier than OCT and detect further damage when the disease has progressed severely. All current data point to a reduced VD in the macular or peripapillary area, or both. As to whether the SVP or DVP is affected more, the results are still vague. Enlargement of the FAZ also seems to be a feature of ON MS. High levels of disability are related to low macular SVP VD and low-contrast visual acuity. Additionally, OCT-A could help in the differential diagnosis between MS and NMOSD with the latest information suggesting a significant reduction in the peripapillary and macular VD and FAZ size in NMOSD-ON compared to MS. Finally, OCT-A may be able to detect disease progression when all other methods, such as OCT, radiological, and even clinical examination, fail. Table 1 summarises all significant findings of OCT and OCT-A in MS. 

## 5. Conclusions

As MS is the most common demyelinating disease of the CNS, it can severely affect patients’ lives by causing a wide range of symptoms. The use of OCT and OCT-A has already been established for the diagnosis and monitoring of several different ophthalmologic conditions. In recent years, numerous studies have shed light on their potential use in various neurodegenerative conditions, including MS. Unquestionably, the existing data are still limited, especially regarding the role of OCT-A; however, various biomarkers could function as targets for future research. This review aims to highlight the most important findings available to date and to aid future research by providing a detailed overview of the current literature.

## Figures and Tables

**Table 1:** OCT and OCT-A findings in MS.

OCT	OCT-A
• RNFL and GC-IPL thinning inversely related to disease duration.	• Earlier detection of the disease and disease-progression compared to OCT
• Lower RNFL values suggest increased disability risk	• Reduced VD in both the macular and peripapillary area
• Significantly lower RNFL and GC-IPL values in NMOSD patients compared to MS	• Reduced peripapillary, macular VD and FAZ size in NMOSD-ON patients compared to MS
• Natalizumab may cause lower rates of GC-IPL thinning	• High disability rates relate to reduced macular SVP VD
	• FAZ enlargement in ON MS patients

RNFL: retinal nerve fibre layer; GC-IPL: ganglion cell inner plexiform layer complex; NMOSD: neuromyelitis optica spectrum disorder; VD: vessel density; FAZ: foveal avascular zone, ON: optic neuritis; SVP: superficial vascular plexus.

## Data Availability

Not applicable.

## Abbreviations

AI	Artificial intelligence
BRAO	Branch retinal artery occlusion
CNS	Central nervous system
DVP	Deep vascular plexus
EBV	Epstein-Barr virus
EDSS	Expanded disability status scale
FAZ	Foveal avascular zone
GC-IPL	Ganglion cell inner plexiform layer complex
mGC-IPL	Macular ganglion cell inner plexiform layer
FNβ-1b	Interferon beta
IPL	Inner plexiform layer
MRI	Magnetic resonance imaging
MS	Multiple sclerosis
pwMS	People with multiple sclerosis
NMOSD	Neuromyelitis optica spectrum disorders
OCT	Optical coherence tomography
OCT-A	Optical coherence tomography with angiography
ON	Optic neuritis
PHOMS	Peripapillary hyper-reflective ovoid mass-like structures
RNFL	Retinal nerve fibre layer
RPC	Radial peripapillary capillary
pRPC	Peripapillary radial peripapillary capillary
SVP	Superficial vascular plexus
UVB	Ultraviolet B light
VD	Vessel density
VEP	Visual evoked potential
mVEP	multifocal visual evoked potential
VVD	Volumetric vessel density
